# Multiple omics revealed the growth-promoting mechanism of *Bacillus velezensis* strains on ramie

**DOI:** 10.3389/fpls.2024.1367862

**Published:** 2024-03-27

**Authors:** Xin Wang, Yanzhou Wang, Yafen Fu, Yang Zhai, Xuehua Bai, Tongying Liu, Guang Li, Liangbin Zeng, Siyuan Zhu

**Affiliations:** Institute of Bast Fiber Crops, Chinese Academy of Agricultural Sciences, Changsha, China

**Keywords:** plant growth-promoting rhizobacteria, microbial diversity, bacterial community, metabolomics correlation, ramie

## Abstract

Beneficial bacteria that promote plant growth can shield plants from negative effects. Yet, the specific biological processes that drive the relationships between soil microbes and plant metabolism are still not fully understood. To investigate this further, we utilized a combination of microbiology and non-targeted metabolomics techniques to analyze the impact of plant growth-promoting bacteria on both the soil microbial communities and the metabolic functions within ramie (*Boehmeria nivea*) tissues. The findings indicated that the yield and traits of ramie plants are enhanced after treatment with *Bacillus velezensis* (*B. velezensis*). These *B. velezensis* strains exhibit a range of plant growth-promoting properties, including phosphate solubilization and ammonia production. Furthermore, strain YS1 also demonstrates characteristics of IAA production. The presence of *B. velezensis* resulted in a decrease in soil bacteria diversity, resulting in significant changes in the overall structure and composition of soil bacteria communities. Metabolomics showed that *B. velezensis* significantly altered the ramie metabolite spectrum, and the differential metabolites were notably enriched (*P* < 0.05) in five main metabolic pathways: lipid metabolism, nucleotide metabolism, amino acid metabolism, plant secondary metabolites biosynthesis, and plant hormones biosynthesis. Seven common differential metabolites were identified. Correlation analysis showed that the microorganisms were closely related to metabolite accumulation and yield index. In the *B. velezensis* YS1 and *B. velezensis* Y4-6-1 treatment groups, the relative abundances of *BIrii41* and *Bauldia* were significantly positively correlated with sphingosine, 9,10,13-TriHOME, fresh weight, and root weight, indicating that these microorganisms regulate the formation of various metabolites, promoting the growth and development of ramie. Conclusively, *B. velezensis* (particularly YS1) played an important role in regulating soil microbial structure and promoting plant metabolism, growth, and development. The application of the four types of bacteria in promoting ramie growth provides a good basis for future application of biological fertilizers and bio-accelerators.

## 1 Introduction

Ramie (*Boehmeria nivea*) is considered one of the most significant bast fiber crops in ancient China and is commonly known as “Chinese grass” (Ni et al., 2018). China is the leading country in terms of ramie production. It accounts for over 90% of the global production and has a significant advantage in terms of cultivation area and total output. Additionally, China possesses a vast array of germplasm resources and holds a dominant position in the international market (Rehman et al., 2020; Fu et al., 2023). Ramie is the second largest fiber crop in China after cotton (Liu et al., 2014). According to archaeological evidence, ramie has been used as a textile material for over 4,700 years owing to its strong natural fibers (Liao et al., 2014; Mu et al., 2022). At present, ramie fiber is commonly used in the textile sector due to its capacity to maintain form, minimize creases, and add a glossy sheen to the look of textiles (Kadolph and Langford, 2001). In recent years, ramie has also been used as an unconventional feed for livestock and poultry because of its high content of crude proteins and amino acids (Kipriotis et al., 2015). Therefore, ramie is an important commercial crop for both fiber and feed.

At present, farmers have applied chemical fertilizers and pesticides extensively to achieve higher agricultural yields. However, chemical fertilizers that are not absorbed and utilized by crops enter the soil easily (Gouda et al., 2018), resulting in the destruction of soil microbial community structure, soil degradation and consolidation, decreased productivity, and poor crop quality (Liu et al., 2015). Thus, the challenge in contemporary society lies in minimizing the reliance on artificial chemical pesticides and fertilizers. An effective approach to achieving this goal is by substituting a portion of synthetic fertilizers and pesticides with natural microbiological fertilizers. The most promising microbial biostimulants include arbuscular plant growth-promoting rhizobacteria (PGPR), mycorrhizal fungi (AMF), and *Trichoderma* spp (Ganugi et al., 2022). Inoculating beneficial microorganisms has shown to have biostimulant activity in plants, offering benefits such as stress alleviation (Sangiorgio et al., 2020), growth promotion (Nacoon et al., 2020), and etc.

PGPR are a class of beneficial bacteria that grow in plant rhizosphere soil, promote plant growth, and improve plant resistance (Bashan et al., 2014). Growth-promoting bacteria and their related growth-promoting factors are important for the research and development of biological bacterial fertilizers. The growth promotion of PGPR can be divided into direct and indirect effects (Arruda et al., 2013). The direct growth promotion mechanisms include improved nitrogen uptake (Taulé et al., 2012), improved root phosphorus solubilization (Wu et al., 2012), improved iron production carriers (Kurabachew and Wydra, 2013), and production of phytohormone-like substances (Piromyou et al., 2010). The indirect growth promotion mechanisms refer to the ability of certain microorganisms to inhibit the growth of pathogenic bacteria (Kheirandish and Harighi, 2015), which ultimately results in improved plant growth. This can be achieved through various means, such as suppressing the growth of harmful pathogens or stimulating the plant’s defense mechanisms to fend off insect herbivores (Pineda et al., 2010; Gamalero and Glick, 2011). These mechanisms play a crucial role in biological control and can contribute to the overall health and productivity of plants. PGPR can not only promote plant growth and reduce the use of chemical fertilizers but also may reduce the application of pesticides to a certain extent (Helaly et al., 2022). Studies on plant rhizosphere growth-promoting bacteria have attracted increasing attention (Bhattacharyya and Jha, 2012).

There is a general agreement among researchers about the impact of PGPR on plants, however, there is a lack of in-depth understanding regarding the specific molecular and physiological mechanisms involved in this symbiosis, as well as how plants respond to PGPR in real-world field environments. However, the interrelationships among plant agronomic traits, microbiota, and metabolites remain largely unknown. In recent years, omics techniques have greatly developed, and the integration of multiple omics datasets has paved the way for a more profound comprehension of interactions between plants and microbes. Multi-omics joint analysis has been developed as a new technique. The application of rhizospheric growth-promoting bacteria in ramie production is almost nonexistent. In this study, strains related to ramie growth were identified using large number of screening procedures. Agronomic traits, metabolic mechanisms, and rhizosphere microbial changes in ramie plants treated with the four strains were comprehensively evaluated using a multi-omics analysis method, providing a basis for the development and application of these four strains in the future.

## 2 Matherials and methods

### 2.1 Screening and identification of PGPR

#### 2.1.1 Screening of PGPR

Strain *B. velezensis* JIN4 was originally isolated from branch tissue of kiwifruit “Jintao” variety. *B. velezensis* LYM4-2 strains were isolated from the rhizosphere soil of lotus, and *B. velezensis* YS-1 and *B. velezensis* Y4-6-1 were isolated from the rhizosphere soil of purple yam. First, the soil sample and disinfected plant tissues were homogenized. Thereafter, each homogenate was serially diluted 10^-1^ to 10^-5^, and 100 μl of each dilution was evenly spread on solid Luria-Bertani (LB) plates and incubated at 25°C for 72 h. After incubation, *Bacillus* isolates were randomly isolated from the plates based on colony morphology and identification was performed by stereomicroscopy. Purified single colonies of *Bacillus* spp. were co-cultured with *P. syringae pv. actinidiae* to screen for antagonistic *Bacillus* species. Briefly, freshly grown Pseudomonas cultures were spread on solid LB medium plates. Following adsorption, a 6 mm well was created using a metallic borer and then filled with 100 μl (108 CFU/ml) of the recently cultivated *Bacillus* culture in medium. The plates were then incubated at 25°C for 48 hours to facilitate bacterial culturing.

#### 2.1.2 Identification and Validation of PGPR

To identify the bacteria at the molecular level, 16S rDNA was amplified by PCR using the standard method in the bacterial gene DNA advance kit (Tiangen) (Singh et al., 2015). Genomic DNA was extracted from the strain for subsequent PCR amplification of 16S rDNA using the primers 27F and 1492R. Amplification products were directly sequenced and subjected to BLASTN analysis. Sequence alignment of isolated strains was performed using MEGA 10.0 software. The neighbor-joining algorithm was utilized for clustering, and the Kimura two-parameter model was employed to calculate evolutionary distances. Node support was determined through bootstrapping, with 1,000 replicates conducted for estimation.

#### 2.1.3 Biochemical characterization of PGPR

Biochemical analyses such as Gram staining, starch agar, indole, methyl red, the Voges–Proskauer test, IMViC test for citrate utilization, and catalase activity were conducted as per the procedures outlined in Prescott and Harley (2002). A carbohydrate utilization test kit (KB 009; HiMedia, India) was employed to assess the bacteria’s ability to use different carbon sources. For the evaluation of cellulose-degrading capabilities, bacterial isolates were streaked on Congo Red agar medium, as described by Gupta et al. (2012). The presence of zones of clearance surrounding and beneath the colonies indicated enzymatic breakdown of cellulose.

The test organism was cultivated in peptone water for 48 hours at 37°C before Nessler’s reagent (0.5 ml) was added to each tube to detect the presence of ammonia production. A brown-to-yellow color indicated a positive result for ammonia production, as described by Cappuccino and Sherman, 1992. Indole-3-acetic acid (IAA) production was also evaluated using a colorimetric method outlined by Bric et al., 1991. To assess phosphate-solubilizing activity, the test isolate was grown on NBRIP medium with tricalcium phosphate, following the method by Mehta and Nautiyal, 2001. The presence of a halo zone around bacterial growth on NBRIP-agar plates, as explained by Frey-Klett et al. (2005), confirmed positive results for phosphate solubilization activity.

### 2.2 Experimental design and sample collection

Activated bacteria were suspended in sterile Nutrient Broth (NB) liquid medium and adjusted to the appropriate concentration. First, the soil for potting studies was autoclaved at 121°C for 1 h, and each plastic pot was filled with 300 g of sterilized soil. Thereafter, 4-weeks-old seedlings were sown in pots (one seedling per pot). Each seedling was irrigated with 15 mL of resuspended bacterial solution at an OD value of 2.5, and sterilized NB liquid medium was used as a control. The bacteria were used twice a week for a total of three weeks. The study utilized a fully randomized layout with six repetitions for each treatment: (i) sterilized LB broth control, (ii) JIN4, (iii) YS1, (iv) Y4-6-1, and (v) LYM4-2. Over a span of 4 weeks, plants were cultivated in a greenhouse under an average daytime and nighttime temperature of 25 and 18°C, respectively, with tap water supplementation as necessary. Throughout the experiment, each treatment was evaluated for its effects on the growth and development of the plants. The data collected was analyzed to determine any significant differences between the treatments. In this study, the ramie growing soil is a nutrition-growing seedling medium (Xiangzhneg Agriculture Technology, PH:5.5-7.0, organic matter ≥20%) purchased by the laboratory, which is very easy to cultivate ramie.

Following the completion of the experiment, a total of six pots from each experimental group were selected at random for analysis. The measurements taken included the length of their stems, the diameter of their stems, as well as their fresh weight and root weight. Additionally, samples of fresh leaves were collected to evaluate the levels of photosynthetic pigments and antioxidants present. The content of relative chlorophyll was determined utilizing a SPAD-502 device (KONICA MINOLTA SENSING, INC., JAPAN) chlorophyll meter (Yu et al., 2016). The activities of catalase (CAT), superoxide dismutase (SOD) and peroxidase (POD) were measured using the detection kit of Solarbio Ltd.

The rhizosphere soil of ramie (six pots for each strain) was sampled using the nomenclature of samples based on the name of the strain (e.g., for JIN4, samples were named JIN4-1, JIN4-2, JIN4-3, JIN4-4, JIN4-5, and JIN4-6), and root irrigation with sterilized NB liquid medium was used as a control (CK). Each sample was analyzed in 6 replicates, and 30 soil samples underwent sieving with a 2-mm sieve, homogenization and storage at −70°C for biological and biochemical analyses. To ensure make the experimental results were representative, six replicate samples of each treatment were measured separately. Similarly, the control and four bacteria-treated whole seedlings were sampled for metabolome analysis.

### 2.3 Microbial diversity analysis

Bacterial DNA was isolated from the 30 soil samples stored in −70°C refrigerator using a MagPure Soil DNA LQ Kit (Magen, Guangdong, China) following the manufacturer’s instructions. The V3-V4 region of the bacterial 16S rRNA gene was amplified using the respective primer pairs 343F and 798R. Sequencing libraries were constructed using the TruSeq^®^ DNA PCR-Free Sample Preparation Kit according to the manufacturer’s instructions. Sequencing was performed on an Illumina NovaSeq6000 platform with two paired-end read cycles of 250 bases each. The 16S rRNA gene amplicon sequencing and analysis were conducted by OE Biotech Co., Ltd. (Shanghai, China). The raw data of 16SrRNA gene sequencing were analyzed using QIIME2 platform (v2020.2). The final effective labels were obtained by splicing, filtering, and removing chimeric sequences using FLASH (version 1.2.7), followed by cluster analysis. Microbial diversity and richness were assessed by calculating the number of OTUs and alpha diversity indexes like Shannon and Chao1. To identify soil microbial community structure and estimate beta diversity, principal component analysis (PCA) was utilized.

### 2.4 Metabolomic analysis and statistical analysis

Liquid chromatography-mass spectrometry (LC-MS) is an analytical instrument that integrates liquid chromatography with mass spectrometry, enabling the effective separation and analysis of intricate organic mixtures. The buds obtained by 2.2 were placed in liquid nitrogen for quick freezing and transferred to a refrigerator at − 70° C for storage.

Freeze-dried shoots of the CK, JIN4, YS1, Y4-6-1, and LYM4-2 plants were triturated in a mixer mill (MM 400, Retsch) with zirconia beads for 1.5 min at 30 Hz. Each approximately 100 mg sample was then sonicated for 30 minutes in 1 mL of pre-cooled solvent (methanol/water 1:1, v/v, containing L-2-chlorophenylalanine, 2 μg/mL). Subsequently, the samples were incubated at -20°C for 20 minutes, followed by centrifugation at 4°C and 13000 rpm for 10 minutes. After that, 150 μL of the resulting supernatant was extracted, passed through a 0.22 μm filter, and transferred to a vial for LC-MS analysis. Six biological replicates were analyzed for each sample. The LC-MS analysis was conducted by injecting a 10 μL sample into an HSS T3 C18 column (100 mm×2.1 mm×1.8 μm, Waters) that was maintained at 50°C. The gradient elution program consisted of the following steps: 0-2 min, 100% A; 2-11 min, 0%-100% B; 11-13 min, 100% B; 13-15 min, 0%-100% A. The Q-TOF mass spectrometer was used in both positive and negative ion modes, with specific parameters like the ESI source temperature set at 120°C and the desorption temperature at 450°C. The LC-MS metabolic profiles were generated through the combination of an ACQUITY UHPLC system (Waters Corporation, Milford, USA) and an AB SCIEX Triple TOF 5600 system (AB SCIEX, Framingham, MA, USA) in ESI positive and negative ion modes. Quality control (QC) samples were included at regular intervals to ensure reproducibility, and metabolites were identified using various databases and tandem mass spectrometry (MS/MS) spectra. Raw LC-MS data were provided by Luming (Shanghai, China). Metabolites were analyzed primarily using RT m/z and tandem mass spectrometry (MS/MS) pairs as well as HMDB and lipid mass spectrometry. The Excel file containing the data matrix, which includes 3D datasets of m/z, RT peaks, and intensities, was exported for additional analysis. PCA and OPLS-DA were performed to display metabolic shifts in the various groups. Metabolites that met the criteria of |log2 fold change|≥1.0 and VIP ≥1.0 were flagged as differentially regulated between the experimental and control groups. These metabolites were compared with the KEGG pathway database to investigate their involvement in distinct metabolic pathways.

### 2.5 Data availability

The datasets from this study have been made available in online repositories. The specific names of the repositories and the accession numbers can be accessed at https://www.ncbi.nlm.nih.gov/GenBank. The *B. velezensis* data set was deposited in the NCBI Sequence Read Archive under accession no. MZ277421.1(JIN4), OP493231.1(YS1), OP493232.1(Y4-6-1), and OP493233.1(LYM4-2).

## 3 Results

### 3.1 Isolation, biochemical trait, and identification

**Figure 1:**
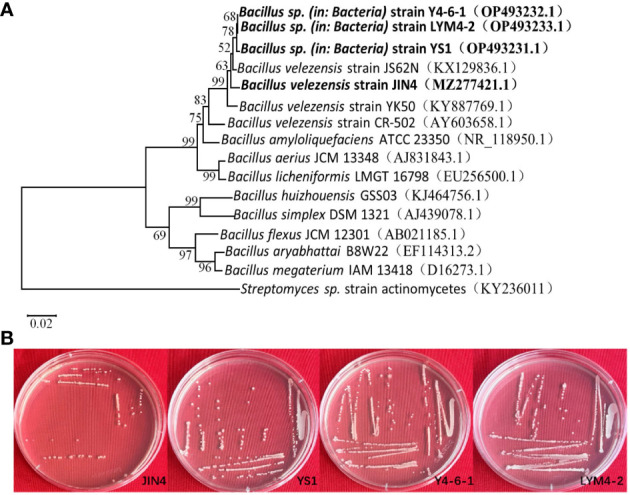
Phylogenetic tree showing the relationship of four *B*. *velezensis* strains with closely related bacteria **(A)**. The 16S rRNA gene sequences of closely related species were downloaded from the NCBI GenBank database. 16S rRNA gene of *Streptomyces* sp. strain actinomycetes was selected as an outgroup. The tree was obtained using neighbor joining method of software package Mega version 10.0 at the bootstrap value of n = 1000. Morphological picture of four *Bacillus velezensis* **(B)**.

Based on colony morphology, *Bacillus* isolates were recovered from the soil and tissue, and an antagonism test was performed. The results showed that the test organism JIN4, YS1, Y4-6-1, and LYM4-2 inhibited the growth of *P. syringae* pv*. actinidiae*. Basic microbiological and biochemical tests of the four isolates indicated that they were Gram-positive bacteria, which showed positive results for methyl red, Voges–Proskauer, catalase, oxidase, lecithinase, gelatin liquefaction, and nitrate reductase, and negative results for indole and hydrogen sulfide. The four test strains were able to utilize various carbon sources, including malanate, citrate, glucose, D-mannose, D-xylose, and D-fructose (**Table 1**). The four test organisms formed clear colony zones on cellulose Congo Red agar media, indicating cellulose enzymatic degradation activity. To ascertain the taxonomic affiliation of the four strains at the molecular level, they were subjected to 16S rRNA gene sequence analysis, which revealed the closest match to the 16S rRNA gene sequence of *B. velezensis* (**Figure 1**). This indicated that all isolates were highly homologous to *B. velezensis*. The obtained sequences were submitted to NCBI GenBank under accession numbers MZ277421.1(JIN4), OP493231.1(YS1), OP493232.1(Y4-6-1), and OP493233.1(LYM4-2).

**Table 1:** Biochemical and physiological characteristics of the four bacterial strains.

Characteristic(s)	JIN4	YS-1	Y4-6-1	LYM4-2
Gram test	+	+	+	+
Indole	−	−	−	−
MR	+	+	+	+
VP	+	+	+	+
Catalase	+	+	+	+
Oxidase	+	+	+	+
Lecithinase	+	+	+	+
Gelatin liquefaction	+	+	+	+
Nitrate reductase	+	+	+	+
Hydrogen sulfide	−	−	−	−
Malanate utilization	+	+	+	+
Citrate utilization	+	+	+	+
Glucose	+	+	+	+
D-galactose	−	−	−	−
D-arabinose	−	−	−	−
D-mannose	+	+	+	+
D-xylose	+	+	+	+
D-fructose	+	+	+	+
Cellulose-degrading	+	+	+	+

**+**, means that the characteristic reaction is positive; -, means that the characteristic reaction is negative. The same as below.

### 3.2 Plant growth-promoting traits

Four *B. velezensis* strains tested positive for ammonia production (**Table 2**). Regarding phytohormone production, strain YS1 was positive for IAA production, whereas the other three strains were negative. Furthermore, a distinct clear zone was observed on a solid agar medium enriched with an insoluble form of phosphate (tricalcium phosphate) by the four *B. velezensis* species, demonstrating their ability to solubilize mineral phosphate.

**Table 2:** Plant growth promoting traits of the four *B. velezensis* strains.

Activity	JIN4	YS1	Y4-6-1	LYM4-2
Ammonia Production	+	+	+	+
IAA	−	+	−	−
Phosphate solubilization	+	+	+	+

### 3.3 Plant growth promotion experiments in the glasshouse

**Figure 2:**
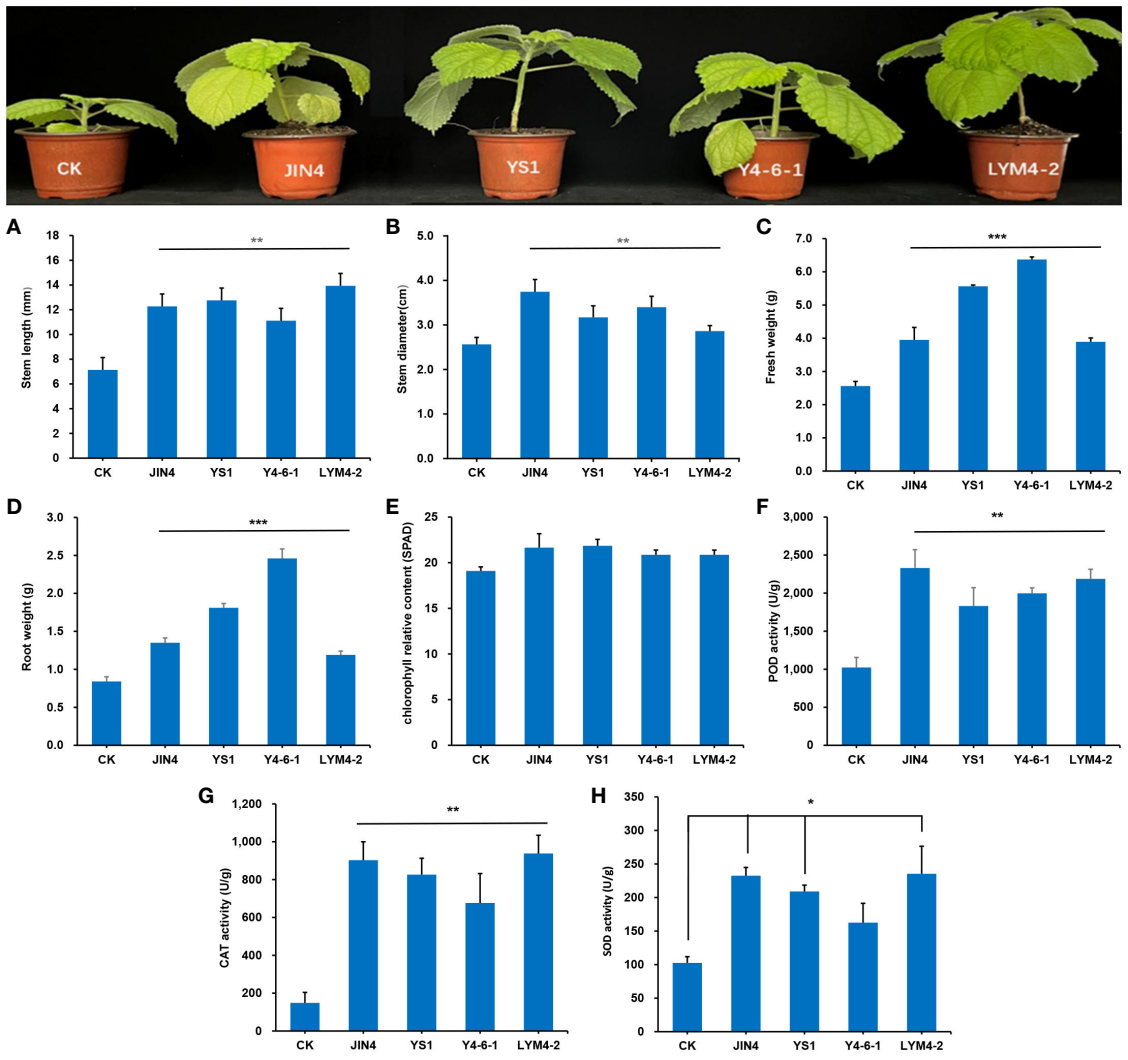
Effects of treatment with four *B*. *velezensis* strains. **(A)** Stem length, **(B)** stem diameter, **(C)** root weight, **(D)** fresh weight, **(E)** chlorophyll relative content, **(F)** POD activity, **(G)** CAT activity, **(H)** SOD activity. * *P* < 0.05, ** *P* < 0.01, *** *P* < 0.001.

The growth status of ramie after four different *B. velezensis* treatments is shown in **Figure 2**. To evaluate the effect of the four *B. velezensis*-treated plants, an analysis of variance was performed (ANOVA; *P* < 0.05), which demonstrated that the inoculation of the four *B. velezensis* resulted in a significant increase in ramie plant growth. Growth was assessed by measuring stem length, stem diameter, fresh weight, root weight, relative chlorophyll content, and antioxidant enzyme activities (POD, CAT, and SOD). After treatment with four bacteria, the stem length was significantly increased by 74%, 77%, 55% and 101%, respectively, compared with the uninoculated plants (*P* < 0.01). Stem diameter also increased significantly by 48%, 27%, 34% and 12%, respectively (*P* < 0.01). Similarly, fresh weight and root weight increased significantly (*P* < 0.001) by 67%, 141%, 229%, 50% and 52%, 115%, 143% and 45%, respectively. The relative chlorophyll content in the four bacterial treatment groups increased, but there was no significant difference compared to that in the control group (*P* > 0.05). The responses of plant antioxidant enzymes to the four *B. velezensis* strains are shown in **Figure 2**. Compared with the control group, the antioxidant enzyme activities of the four bacterial treatment groups significantly increased (*P* < 0.01), in addition to the SOD activity of the Y4-6-1 treatment.

### 3.4 Richness and diversity analysis of ramie soil microbial community

**Figure 3:**
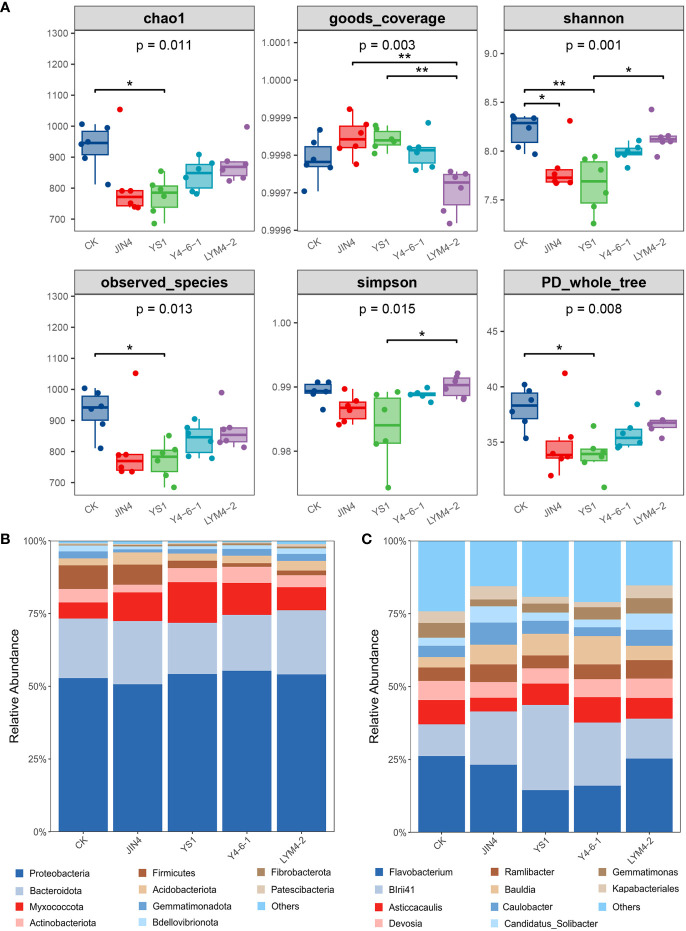
Richness and diversity analysis of ramie soil microbial community **(A)**; Relative abundances of dominant bacterial phyla **(B)** and genera **(C)** after treatment with the four *B*. *velezensis* strains. * *P* < 0.05, ** *P* < 0.01.

The results showed that alpha diversity and richness indices (Chao1, Shannon) were significantly decreased in the JIN4 and YS-1 groups compared to the (control) CK group, and the other groups showed no significant differences compared to the CK group (**Figure 3A**). In summary, the application of rhizosphere growth-promoting bacteria changed the diversity and richness indices of the microbial community in ramie soil.

### 3.5 Bacterial relative abundance and composition in the ramie soil

The bacterial sequences were distributed across 29 phyla, 315 families, and 612 genera (**Supplementary Data 2**). The dominant phyla in the bacterial communities across all soil samples were Proteobacteria (average per sample: 53.38%), *Bacterioidetes* (20.20%), *Myxococcota* (9.63%), *Actinobacteria* (4.36%), and *Firmicutes* (4.08%), which represented more than 80% of all sequences (**Figure 3B**). Compared with control (CK), among the top 10 phyla, the relative abundance of *Myxococcota* significantly increased following treatment with the four *B. velezensis* strains, but Firmicutes were significantly lower in the YS1, Y4-6-1, and LYM4-2 treatment groups (*P* < 0.01). In addition, the relative abundance of Proteobacteria significantly increased following treatment with *Bacillus* Y4-6-1 (*P* < 0.01). However, the relative abundances of *Bacterioidetes* and *Actinobacteriota* significantly decreased following treatment with *Bacillus* YS1 (*P* < 0.01) and *Bacillus* JIN4 (*P* < 0.05), respectively. In summary, treatment with *B. velezensis* strains significantly changed the diversity and richness indices of the microbial community.

The top 10 genera of bacteria in all soil samples were *Flavobacterium* (7.20%), *BIrii41* (6.70%), *Asticcacaulis* (2.52%), *Bauldia* (2.31%), *Devosia* (2.09%), *Ramlibacter* (1.84%), *Caulobacter* (1.70%), *Steroidobacter* (1.61%), *Curvibacter* (1.43%), and *Gemmatimonas* (1.37%) (**Figure 3C**), which together represented less than 30% of all sequences. Compared with the control (CK), there were significant differences in the relative abundances of the top 10 genera (*P* < 0.05). The abundance of *Flavobacterium* in the YS1 and Y4-6-1 groups decreased significantly by 32.44% and 25.91% (*P* < 0.01), respectively. No significant changes were noted in the abundance of the *BIrii41* and *Bauldia* in the LYM4-2 group, whereas the abundance of *BIrii41* in the JIN4, YS1, and Y4-6-1 groups increased significantly (*P* < 0.01). In addition, the abundance of *Ramlibacter* showed differences among the different treatment groups; the YS1 group showed no significant changes, whereas the JIN4, Y4-6-1, and LYM4-2 groups showed significant decreases of 50.30%, 35.45%, and 47.82%, respectively. These results indicate that the relative content and diversity of bacteria at the genus level in the ramie rhizosphere changed significantly after the application of *B. velezensis*.

Except for differences at the phylum and genus levels, certain bacteria showed increased abundance in the five treatments. A total of 32 biomarkers were detected at different taxonomic levels, including phylum, family, class, order, genus, or species, with a log10 LDA score greater than 4.0. These biomarkers reflected distinct species variations among the treatments (**Supplementary Figure S1**). In addition, enrichment of *B. velezensis* in each treatment group was identified at the species level. Compared with the control group, other groups had enrichment of *B. velezensis*; the LYM4-2 group was the most enriched (**Supplementary Figure S1**).

### 3.6 Metabolic differences in different ramie treatments

In order to assess how various *B. velezensis* strains affect the metabolic processes in ramie, metabolomics analysis was performed on differently treated ramie samples using an LC-MS platform. Following quality control (QC), PCA was utilized to compare the variations among the five experimental groups (**Supplementary Figure S2A**). The QC samples exhibited close clustering, indicating the reliability and accuracy of the analysis method, resulting in high-quality data. In total, compared with the control, we detected 455 known differential metabolites (DMs) from 4 different treatments. These included lipids and lipid-like molecules (34.5%); organooxygen compounds (14.7%); organic oxygen compounds (14.1%); organoheterocyclic compounds (4.6%); phenylpropanoids and polyketides (13.0%); benzenoids (9.0%); nucleosides, pyrimidine nucleotides, nucleotides, and analogues (4.2%); organic nitrogen compounds (2.6%); alkaloids and derivatives (1.3%); lignans, neolignans and related compounds (1.1%); and other unclassified metabolites.

Multivariate statistical methods were employed to investigate the significant intergroup correlation present in the study. Through the application of supervised discriminant analysis (PLS-DA), differences within and between the control and treatment groups were thoroughly examined. This allowed for a comprehensive assessment of the impact of various *B. velezensis* strains on the metabolites present in ramie tissue. Based on the PLS-DA (**Figures S2B–E**), the metabolite compositions were comparable among the four bacterial treatments, but all differed from the control group. The PLS-DA model indicated that it was suitable for screening DMs (R_2_Y > 0.95, Q_2_Y < 0.19) (**Supplementary Figure S3**), which showed variations between the treatment and control groups. Volcano plots were employed to screen the discriminative metabolites (**Supplementary Figure S4**).

**Figure 4:**
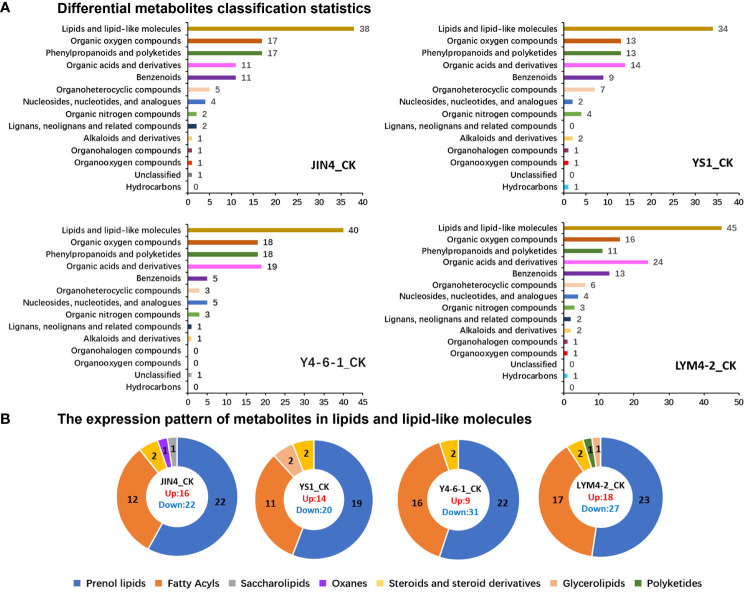
Analysis of the expression patterns of DMs. **(A)** DMs classification statistics. **(B)** The expression pattern of lipids and lipid-like molecules.

Hierarchical clustering analysis was carried out on all DMs within all pairs of comparisons. Various treatments of *B. velezensis* exerted distinct impacts on metabolites present in ramie tissues. As depicted in **Figure 4A**, the DMs generated by plant leaves subjected to different *B. velezensis* treatments predominantly comprised lipids and lipid-like compounds (34–45), with their atypical metabolism potentially influencing plant growth and development. Other metabolites produced included phenylpropanoids and polyketides (11–18), organic acids and derivatives (11–24), and organic oxygen compounds (13–18). Lipids and lipid-like molecules were further classified and analyzed using pie charts as follows: JIN4_CK (16 upregulated, 22 downregulated), YS1_CK (14 upregulated, 20 downregulated), Y4-6-1_CK (9 upregulated, 31 downregulated), and LYM4-2_CK (18 upregulated and 27 downregulated). The major DMs were prenol lipids (19–23), fatty acids (11–17), and steroids and steroid derivatives (2–2) (**Figure 4B**).

**Figure 5:**
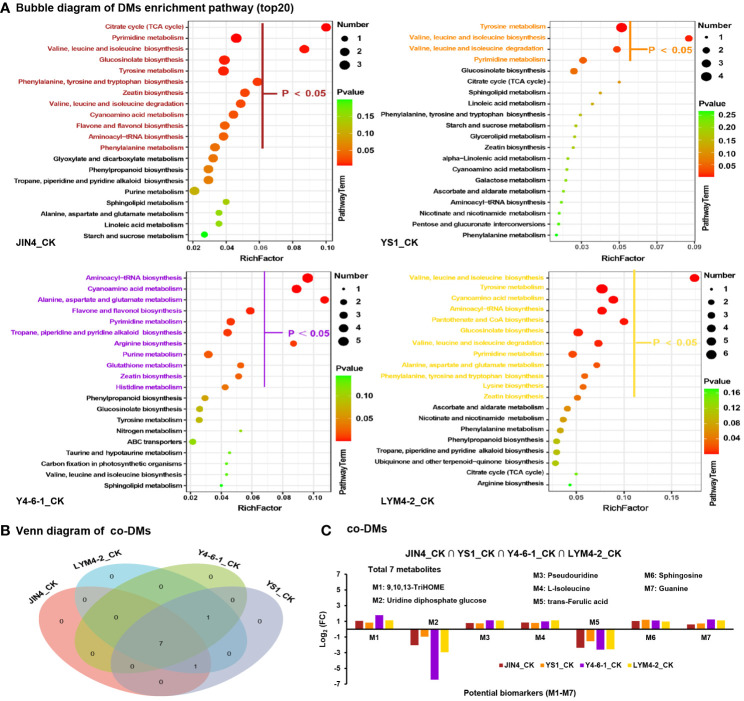
Analysis of metabolic pathways for DEMs. **(A)** Enrichment analysis of KEGG pathways of metabolites with significant differences in abundance among treatments. The X-axis displays the ratio of DEMs within the pathway to the total number of identified metabolites. Higher ratios indicate greater enrichment of DEMs in the pathway. Point color indicates the P-value from the hypergeometric test, with smaller values indicating higher reliability and statistical significance. Dot size corresponds to the number of DEMs within each pathway; larger dots represent more DEMs. **(B)** Evaluation of co-expressed DEMs across diverse treatment conditions. **(C)** Enrichment of co-expressed DEMs in metabolic pathways based on the average expression ratio between sample groups (Log2 FC). Positive values signify upregulation, while negative values indicate downregulation.

Enrichment analysis of KEGG pathways (top 20) indicated that all DMs were enriched in several key pathways including glucosinolate biosynthesis; pyrimidine metabolism; cyanoamino acid metabolism; tyrosine metabolism; zeatin biosynthesis; valine, leucine, and isoleucine biosynthesis; and aminoacyl-tRNA biosynthesis. This suggests that the treatment with *B. velezensis* had a significant impact on the synthesis of metabolites and their corresponding metabolic pathways, as shown in **Figure 5A**. A total of 7 common differentially expressed metabolites (DEMs) were identified by mapping to the KEGG metabolic pathway database, which could be used as potential biomarkers (**Figure 5B**). The DEMs involved in the metabolic pathways included 9,10,13-TriHOME (C_18_H_34_O_5_), uridine diphosphate glucose (C_15_H_22_N_2_Na_2_O_17_P_2_), pseudouridine (C_9_H_12_N_2_O_6_), L-isoleucine (C_6_H_13_NO_2_), trans-Ferulic acid (C_10_H_10_O_4_), sphingosine (C_18_H_37_NO_2_), and guanine (C_5_H_5_N_5_O) (**Figure 5C**).

**Figure 6:**
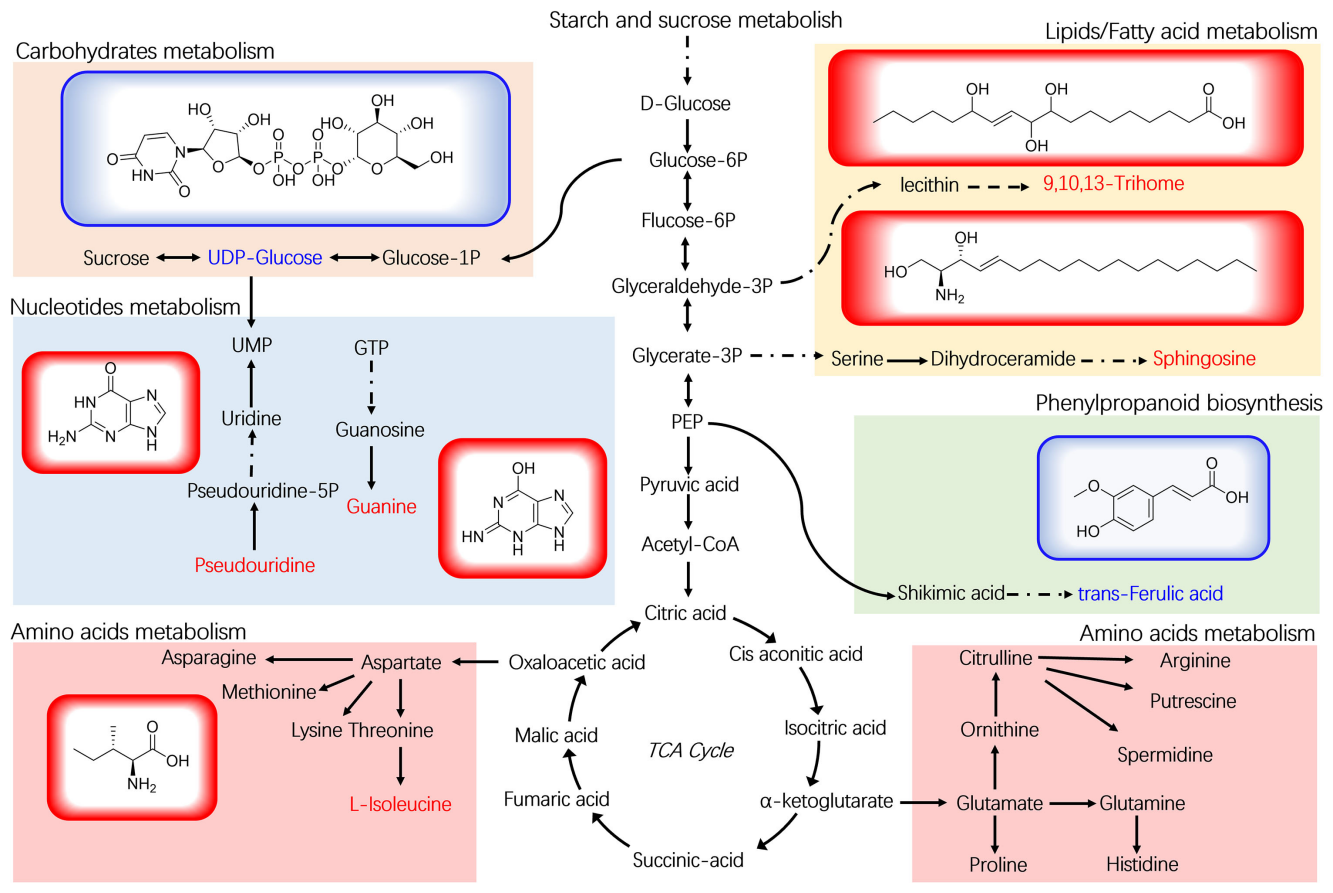
Plant metabolomic analysis of ramie treated with *B*. *velezensis*. The structural formulas of 7 DEMs are shown, along with the metabolic pathway map of differential marker metabolites. UDP-Glucose: uridine diphosphate glucose.

To gain a deeper understanding of how *B. velezensis* influences metabolism in ramie plants, we constructed a metabolic pathway consisting of 7 DEMs through research of the KEGG pathway database (**Figure 6**). These metabolites are involved in the metabolism of lipids, nucleotides, and amino acids, as well as the biosynthesis of plant secondary metabolites and plant hormones. In the four different *Bacillus* treatment groups, the trends for the seven metabolites were consistent. Compared with the control, the contents of the metabolites 9,10,13-TriHOME, pseudouridine, L-isoleucine, sphingosine, and guanine increased significantly in the four *B. velezensis* treatments. However, the levels of uridine diphosphate, glucose, and trans-Ferulic acid decreased significantly after the four different *B. velezensis* treatments. These findings suggest that *B. velezensis* has an indirect impact on the accumulation and makeup of plant metabolites. The irregular expression of these compounds could be a contributing factor to the enhanced growth of ramie following treatment with *B. velezensis*.

### 3.7 The relationship between soil microorganisms, plant metabolites, and ramie agronomic characters

Elucidating the relationships between microbes, metabolites, and yield indices is necessary to optimize PGPR for maximal plant growth. Therefore, the correlations between ramie yield indices, rhizosphere microorganisms, and metabolites were analyzed; their correlation coefficients (cor) were calculated, and the relationships were visualized, as shown in **Table 3**. Analysis of correlation revealed a circular relationship among the three variables (*P* < 0.05). Significantly strong correlations (*P* < 0.05) were evident among the top 10 bacterial genera, DEMs, and yield indices. The findings indicated that the relative abundances of three bacterial genera (*BIrii41*, *Bauldia*, and *Ramlibacter*) had the strongest correlations with certain metabolites (Sphingosine, 9,10,13-TriHOME, and trans-Ferulic acid) and yield indices (fresh weight, root weight, and stem length), indicating the role of these microbes in stimulating the growth and development of ramie through the regulation of metabolite production.

**Table 3:** The correlation analysis between ramie yield indices, microorganisms and metabolites.

Var 1	Interraction	Var 2	Cor	*P* value
Fresh weight	Positive	*BIrii41*	0.8536	2.4E-05
Fresh weight	Positive	*Bauldia*	0.8250	0.0002
Root weight	Positive	*BIrii41*	0.8107	0.0004
Root weight	Positive	*Bauldia*	0.7893	0.0007
Root weight	Negative	*Flavobacterium*	-0.7893	0.0007
Fresh weight	Negative	*Flavobacterium*	-0.7679	0.0013
SOD	Positive	*Caulobacter*	0.6315	0.0116
SOD	Negative	*Asticcacaulis*	-0.6064	0.0165
Stem length	Positive	*Bauldia*	0.6036	0.0195
Stem length	Positive	*Ramlibacter*	0.6036	0.0195
SOD	Positive	*Ramlibacter*	0.5939	0.0196
CAT	Positive	*Caulobacter*	0.5714	0.0286
CAT	Negative	*Asticcacaulis*	-0.5357	0.0422
*Ramlibacter*	Positive	Pseudouridine	0.6979	0.0000
*Ramlibacter*	Positive	Guanine	0.6507	0.0001
*Ramlibacter*	Positive	9,10,13-TriHOME	0.6214	0.0003
*Bauldia*	Positive	9,10,13-TriHOME	0.6027	0.0004
*Ramlibacter*	Negative	Uridine diphosphate glucose	-0.5443	0.0019
*Ramlibacter*	Positive	L-Isoleucine	0.5506	0.0019
*Ramlibacter*	Negative	trans-Ferulic acid	-0.5395	0.0024
*Bauldia*	Positive	Pseudouridine	0.5284	0.0027
*Bauldia*	Positive	Guanine	0.5249	0.0029
*Bauldia*	Negative	Uridine diphosphate glucose	-0.4869	0.0064
*Bauldia*	Positive	Sphingosine	0.4761	0.0078
*BIrii41*	Positive	Sphingosine	0.4710	0.0093
*Bauldia*	Positive	L-Isoleucine	0.4332	0.0168
*Bauldia*	Negative	trans-Ferulic acid	-0.4136	0.0231
*BIrii41*	Positive	Guanine	0.4136	0.0239
*BIrii41*	Positive	Pseudouridine	0.3967	0.0308
*BIrii41*	Positive	9,10,13-TriHOME	0.3882	0.0348
*BIrii41*	Positive	L-Isoleucine	0.3740	0.0425
Sphingosine	Positive	Fresh weight	0.8000	0.0005
9,10,13-TriHOME	Positive	Stem length	0.7464	0.0021
trans-Ferulic acid	Negative	Stem length	-0.7214	0.0033
Sphingosine	Positive	Root weight	0.6857	0.0062
9,10,13-TriHOME	Positive	Root weight	0.6786	0.0069
9,10,13-TriHOME	Positive	Fresh weight	0.6643	0.0086
Uridine diphosphate glucose	Negative	Root weight	-0.5845	0.0221
L-Isoleucine	Positive	CAT	0.5786	0.0264
L-Isoleucine	Positive	Stem length	0.5750	0.0275
trans-Ferulic acid	Negative	Root weight	-0.5750	0.0275
Guanine	Positive	Fresh weight	0.5714	0.0286
trans-Ferulic acid	Negative	Fresh weight	-0.5714	0.0286

## 4 Discussion

In this study, NovaSeq sequencing and LC-MS were employed to analyze the variations in bacterial communities and metabolomics in ramie soil resulting from various treatments of *B. velezensis*. Soil microorganisms are essential components of microbial communities that have significant impacts on nutrient cycling, soil characteristics, plant development, and the overall sustainability of ecosystems (Zhang Z. et al., 2019). Different treatments with *B. velezensis* altered the diversity and abundance index of ramie rhizosphere soil and the metabolite composition and content of the ramie tissue. It was demonstrated that the four PGPR bacteria alter the rhizosphere microbiome, thereby regulating plant tissue metabolism and promoting plant growth by altering the abundance of other beneficial bacteria.

Soil microorganisms, particularly bacteria, are highly plentiful and varied, exerting significant influence in agricultural environments by aiding in nutrient cycling, upholding soil integrity, and enhancing plant development (Gans et al., 2005). Specific bacteria capable of promoting plant growth offer plants mechanisms to resist stress. Findings revealed that introducing *B. velezensis* altered the bacterial community diversity in soil ramie by reducing the alpha diversity (Chao1, Shannon) across all four treatment groups. This decrease could be attributed to the preferential growth of advantageous microorganisms driven by root secretions, resulting in the diminished presence of non-beneficial microbes. In the rhizosphere, plants attract soil microbes that are often plant-beneficial bacteria through the release of plant root exudates (Berendsen et al., 2012). The composition of soil bacterial communities is influenced by the application of *B. velezensis* (Sun et al., 2022) and the present results showed similar impacts. It was demonstrated that *B. velezensis* (JIN4, YS1, Y4-6-1, and LYM4-2) increased the abundance of various genera with documented beneficial roles, such as *BIrii41* (Jansen et al., 2008; Meiser, 2008), *Bauldia* (Yee et al., 2010), and *Ramlibacter* (Sun et al., 2019; Zhang L. N. et al., 2019). The promotion of advantageous bacteria in the rhizosphere seems to be a shared trait of *Bacillaceae*. Previous reports have indicated that *B. velezensis* can stimulate native soil Pseudomonas communities, which in turn enhance plant disease suppression (Sun et al., 2022). Furthermore, the secondary compounds generated by *B. velezensis* have the potential to induce systemic resistance in plants and support robust plant growth (Fan et al., 2018).

Plant growth-promoting properties also showed that the JIN4, YS1, Y4-6-1, and LYM4-2 groups produced ammonia and solubilized phosphate. Phosphate solubilization is a crucial characteristic of microorganisms in the rhizosphere, which plays a key role in providing plant growth with bioavailable phosphate (Liu et al., 2014; Dutta et al., 2015). The existence of bacteria that solubilize phosphate in soil can be viewed as a promising sign of using microbial biofertilizers to enhance crop yield and promote sustainable agriculture. Furthermore, apart from its ability to produce ammonia and solubilize phosphate, the isolated strain YS1 displayed additional characteristics that promote plant growth. These include the synthesis of plant hormones, specifically auxins, that support the enhancement of plant productivity. Introducing bacteria that produce IAA stimulates root development through the augmentation of adventitious roots in terms of both quantity and length, along with modifications to root structure. This ultimately improves nutrient absorption and fosters plant growth (Chakraborty et al., 2006; Yang et al., 2009; Dutta et al., 2015).

Relative chlorophyll content can reflect changes in plant photosynthesis after bacterial treatment. In this study, the relative content of chlorophyll in ramie treated with the four bacteria increased to a certain extent, reflecting the health of plant leaves and an increase in photosynthesis. It has been speculated that these four bacterial species are involved in plant growth and metabolism.

The crucial role of the plant antioxidant enzyme system that fights off stress and eliminates free radicals cannot be overstated. Plants respond to adverse conditions by activating defense mechanisms. These mechanisms trigger the production of damaging substances like oxygen free radicals, peroxides, and membrane lipid peroxides, leading to the degradation of cell membrane structure and function (Singh and Jha, 2016). Four unique *B. velezensis* treatments led to a notable surge in antioxidant enzyme levels within plants, bolstering defense mechanisms and fostering unhindered plant development.

Metabolomics can reveal changes in endogenous substances and the molecular regulation mechanisms of organisms in different environments. The response strategies of the plants to the four growth-promoting bacteria were similar. The primary discrepancies found in metabolites were observed in lipids and lipid compounds, organic acids along with their variations, and organic oxygen substances. These variances showcase the enhancement of lipid and carbon metabolism within plants and could provide insights into potential pathways for stimulating plant development. A crucial metabolite, 9,10,13-TriHOME, is formed from linoleic acid oxidation, and is essential for plant defense mechanisms (Zeng et al., 2017). 9,10,13-TriHOME is associated with plant disease resistance and may induce plant root rot resistance by promoting linoleic acid and tyrosine metabolism (Zhang et al., 2022). Sphingolipids are essential components of the plasma membrane and other intramembrane systems, serving not just as structural elements but also as signaling molecules in response to various stresses. Recent research has highlighted the crucial involvement of sphingolipid metabolism in the regulation of plant growth and development (Ali et al., 2018). Among the diverse types of sphingolipids, sphingosine stands out as a key constituent of cellular membranes. In plants, sphingosine functions as a metabolic intermediate of sphingosine-1-phosphate, which in turn plays a pivotal role in enhancing plant resistance against diseases.

Uridine diphosphate glucose serves as a signaling molecule in plants and plays a crucial role in processes such as plant growth, development, and stress response. This molecule is involved in regulating various metabolic pathways including glucose metabolism, carbon metabolism, and phenylpropane metabolism. Furthermore, it impacts the signaling of plant hormones and the communications between plants and pathogens. For example, uridine diphosphate glucose is involved in zeaxanthin metabolism. Zeatin is a naturally occurring cytokinin that promotes cell division and differentiation and regulates plant growth and development. Pseudouridines are commonly present in evolutionarily conserved and functionally crucial sections of rRNA, tRNA, and additional noncoding RNA molecules. Pseuduridine modification helps stabilize RNA structure and ribosome biogenesis and activity and regulates rRNA processing, pre-mRNA splicing, and protein synthesis, thereby controlling growth, development, and response to stress in different organisms (Lu et al., 2017; Adachi et al., 2019; Sun et al., 2019; Wang et al., 2022). Ferulic acid and other phenolic acids have an inhibitory effect on plant growth, which can inhibit plant nutrient absorption, photosynthesis, respiration, the function and activity of various enzymes, endogenous hormone synthesis, and protein synthesis (Hussain and Reigosa, 2021; John and Sarada, 2012).

**Figure 7:**
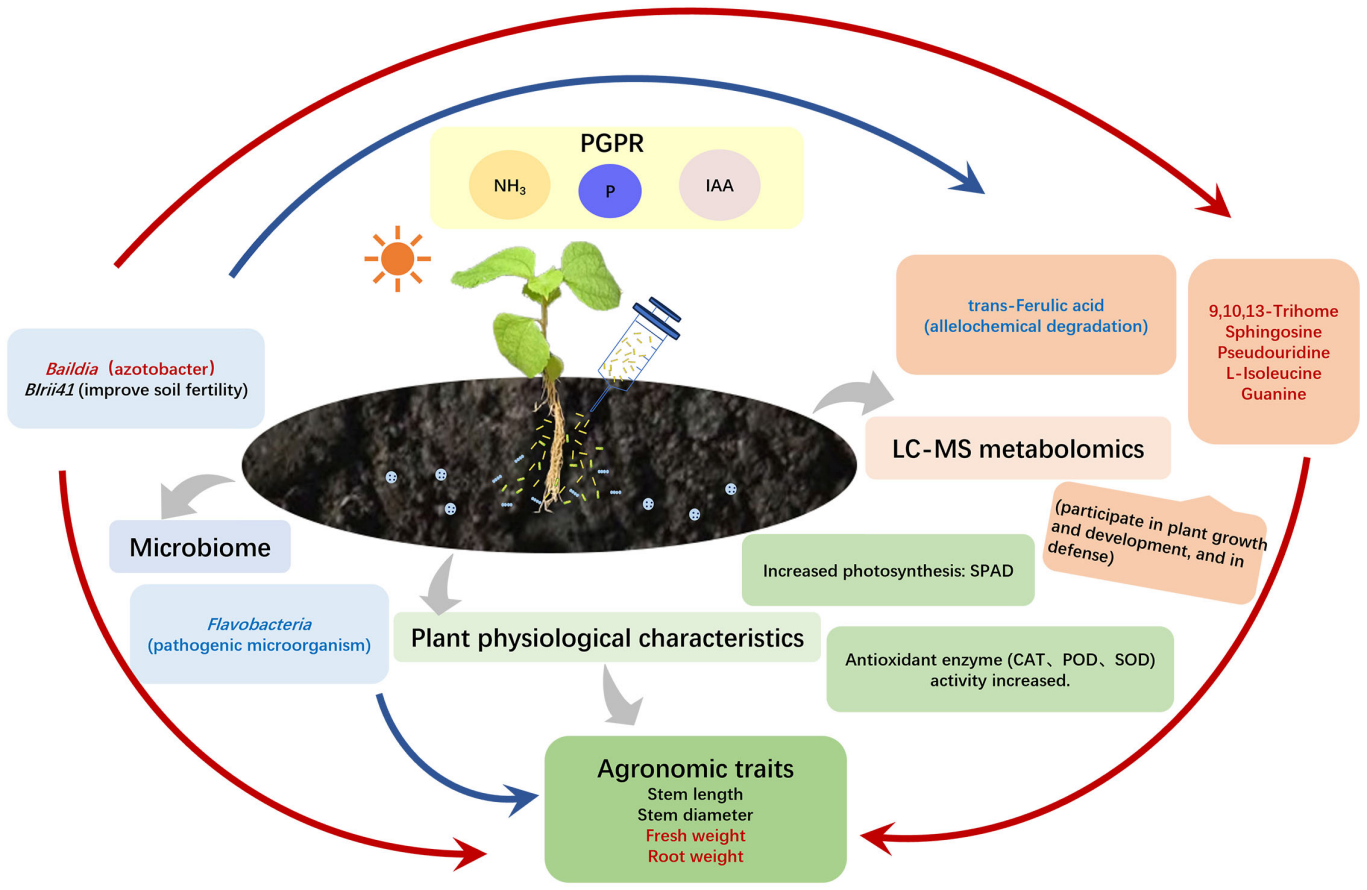
Mechanism diagram of *B*. *velezensis* promoting ramie growth. The red arrow shows a positive relationship, while the blue arrow represents a negative correlation. The red font signifies a noteworthy rise, and the blue font indicates a notable decline.

This study revealed the regulatory microbe–metabolite yield relationship between microorganisms, DEMs, and yield traits at the macro and micro levels, indicating that the rhizosphere microbial composition was dominated by drug-resistant bacteria, *Azotobacter* spp., and degrading bacteria, which had a strong correlation with the synthesis of organic acids and lipid metabolism, promoting an increase in plant fresh weight and root weight. The findings from this research indicated that the levels of *BIrii41* and *Bauldia* significantly rose in the YS1 group and Y4-6-1 group. In contrast, the relative abundance of *Flavobacterium* decreased significantly, corresponding to the significant increases of metabolites 9,10,13-TriHOME, sphingosine, uridine, guanine, and l-isoleucine along with the corresponding root weight and fresh weight of plants. In addition, the increased relative abundance of *Bauldia* significantly reduced uridine diphosphate, glucose, and trans-Ferulic acid contents. *Bauldia* is a nitrogen-fixing bacterium that inhibits plant diseases and promotes plant growth (Black et al., 2012; Kumar et al., 2012). *Myxobacteria*, for example BIrii41_norank, are found extensively throughout the environment and are plentiful in aerobic compost as well as vermicompost (Cai et al., 2018). The secondary metabolites of *Myxobacteria* exhibit antiviral and antifungal properties, capable of inhibiting eukaryotic RNA, DNA, and protein synthesis, as well as disrupting heavy metal ion transport, among other functions (Jansen et al., 2008; Meiser, 2008). *Flavobacterium* is the dominant bacterium in the rhizosphere soil that inhibits soil-borne bacterial wilt and is closely related to the inhibition ability of Verticillium wilt. However, some studies have found that certain *Flavobacterium* species can harm plant health as pathogenic microorganisms (Fu et al., 2018; Bao et al., 2022). Therefore, as shown in **Figure 7**, it is speculated that rhizosphere growth-promoting *B. velezensis* YS1 and Y4-6-1 may recruit beneficial bacteria *BIrii41* and *Bauldia* to reduce harmful *Flavobacterium*, promote plant lipid, carbon, amino acid, and nucleic acid metabolisms, as well as the production of phenolic acid auto-toxic substances, particularly in the YS1 strain, and also promote the production of IAA, thereby improving growth and development (Zhang et al., 2017).

## 5 Conclusion

The relationship between bacterial communities and metabolomics was investigated by utilizing NovaSeq sequencing and LC-MS for the analysis in ramie after different treatments with *B. velezensis*. Based on the diversity index, the four *B. velezensis* strains showed reduced bacterial diversity compared with the control. Furthermore, the four *B. velezensis* strains significantly changed bacterial community structures and compositions. Metabolomics studies showed that YS1 and Y4-6-1 caused significant changes in plant metabolic spectrum and affected metabolic pathways related to lipid metabolism, while *B.velezensis* enhanced the activity of lipid metabolism-related pathways. Additionally, these changes were closely linked to shifts in specific microbial taxa. Metabolites have strong connections with various rhizosphere microbes, which can significantly influence the composition of microbial communities in the rhizosphere of ramie. Through correlation analysis, it was evident that the abundance levels of *BIrii41* and *Bauldia* exhibited a positive relationship with sphingosine, 9,10,13-TriHOME, as well as the fresh weight and root weight. This suggests that these microorganisms play a crucial role in enhancing the growth and development of ramie by modulating the production of most ramie metabolites. The findings of this research validate the interconnectedness between soil microorganisms, plant metabolism, and yield indicators, indicating that the application of *B. velezensis* (particularly YS1) can yield more favorable outcomes. These insights may serve as a valuable guide for implementing microbial strategies to enhance soil quality and crop productivity sustainably.

## Data availability statement

The original contributions presented in the study are included in the article/**Supplementary Material**. Further inquiries can be directed to the corresponding author.

## Author contributions

XW: Writing – original draft, Data curation, Formal Analysis, Writing – review & editing. YW: Conceptualization, Methodology, Writing – review & editing. YF: Data curation, Writing – original draft. YZ: Investigation, Validation, Writing – review & editing. XB: Investigation, Validation, Writing – review & editing. TL: Investigation, Validation, Writing – review & editing. GL: Data curation, Writing – review & editing. LZ: Supervision, Writing – review & editing. SZ: Funding acquisition, Project administration, Supervision, Writing – review & editing.
