# Supplementary material for: Multiple omics revealed the growth-promoting mechanism of Bacillus velezensis strains on ramie
Source: Front Plant Sci. 2024 Mar 27;15:1367862. doi: 10.3389/fpls.2024.1367862 (PMC11004232; doi:10.3389/fpls.2024.1367862)
Supplement: Supplementary file 1 [file DataSheet_1.docx]

**Multiple omics revealed the growth promoting mechanism of *Bacillus velezensis* on ramie**

Xin Wang^a^^†^, Yanzhou Wang^a†^, Yafen Fu^a†^, Yang Zhai^a^, Xuehua Bai^a^, Tongying Liu^a^, Guang Li^a^, Liangbin Zeng^a*^ and Siyuan Zhu^a*^

^†^ These authors have contributed equally to this work and share the first authorship.

*^a^Institute of Bast Fiber Crops, Chinese Academy of Agricultural Sciences, Changsha 410205, China*

^*^**Corresponding author:**

**Liangbin Zeng**

Institute of Bast Fiber Crops,

Chinese Academy of Agricultural Sciences, Changsha 410205, China

E-mail: zengliangbin@caas.cn (L. Zeng).

**Siyuan Zhu**

Institute of Bast Fiber Crops,

Chinese Academy of Agricultural Sciences, Changsha 410205, China

E-mail: zhusiyuan@caas.cn (S. Zhu).

**Figure** **captions:**

**Fig. S1** Results of LEfSe analysis showing bacterial taxa that differed significantly across the five treatments in rhizosphere soil (A). Cladogram plotted from LEfSe analysis showing significant differences (P < 0.05) in relative abundance of 16S rRNA gene-based bacterial taxa of rhizosphere soil (B) across the five treatments. Relative abundances of *B. velezensis* species after four treatments (C).

**Fig. S2** Metabonomic analysis of four *B. velezensis*-treated samples. (A) Principal component analysis (PCA) of metabolites in the rhizosphere soil. (B–E) PLS-DA analysis of metabolites. (F) Analysis of co-expressed DMs in different treatment groups. Refer to Table S1 for detailed annotation information for all metabolites.

**Fig. S3** Metabonomic analysis of four *B. velezensis* treated. (A-D) Cross-validation model of PLS-DA.

**Fig. S4** The expression volcano map of differential metabolites up and down regulate. Blue dots represent down-regulated metabolites, red dots represent up-regulated metabolites, and gray dots represent no-differential metabolites.


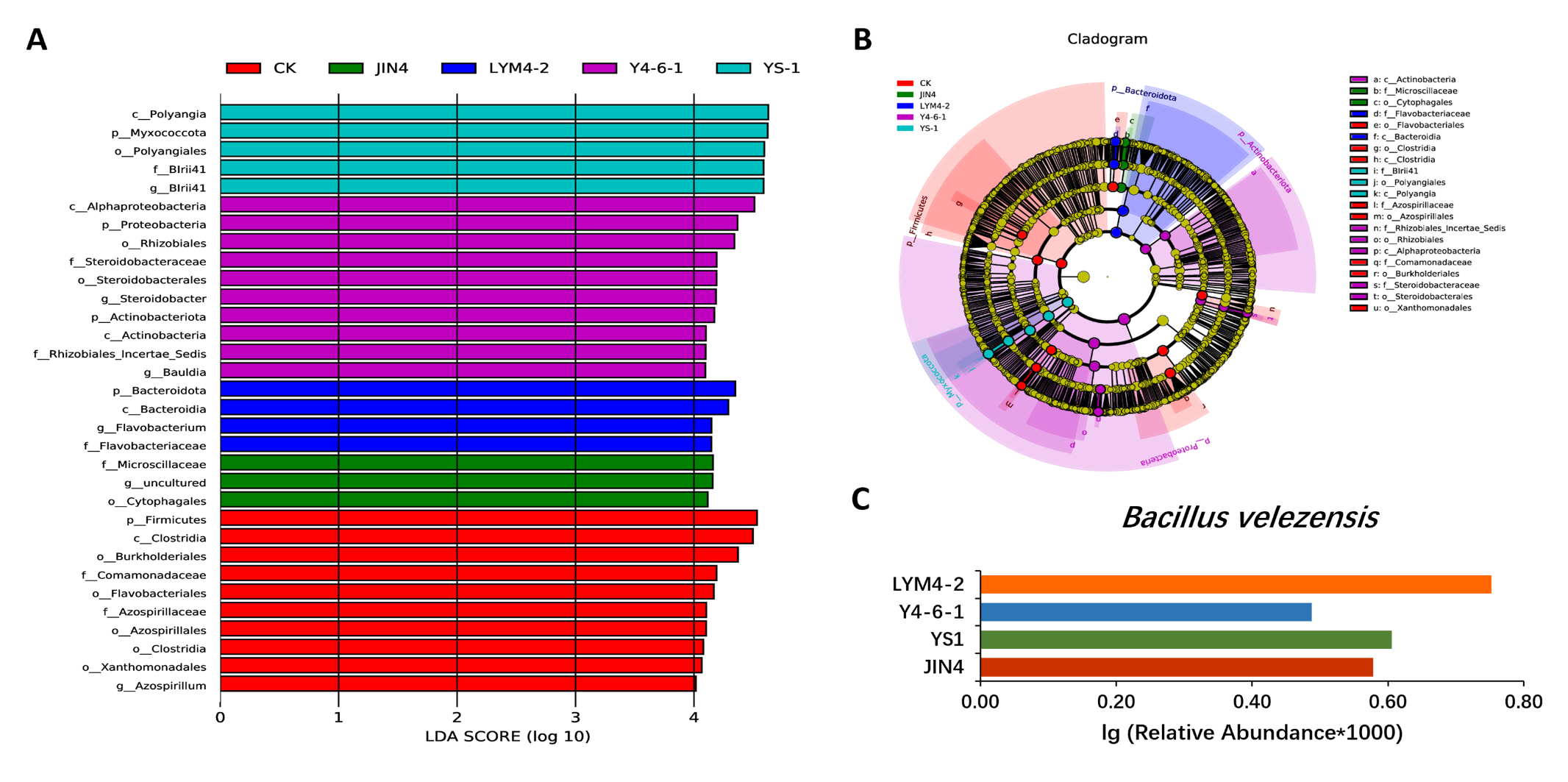


**Fig. S1.** Results of LEfSe analysis showing bacterial taxa that differed significantly across the five treatments in rhizosphere soil (A). Cladogram plotted from LEfSe analysis showing significant differences (*P* < 0.05) in relative abundance of 16S rRNA gene-based bacterial taxa of rhizosphere soil (B) across the five treatments. Relative abundances of *B. velezensis* species after four treatments (C).
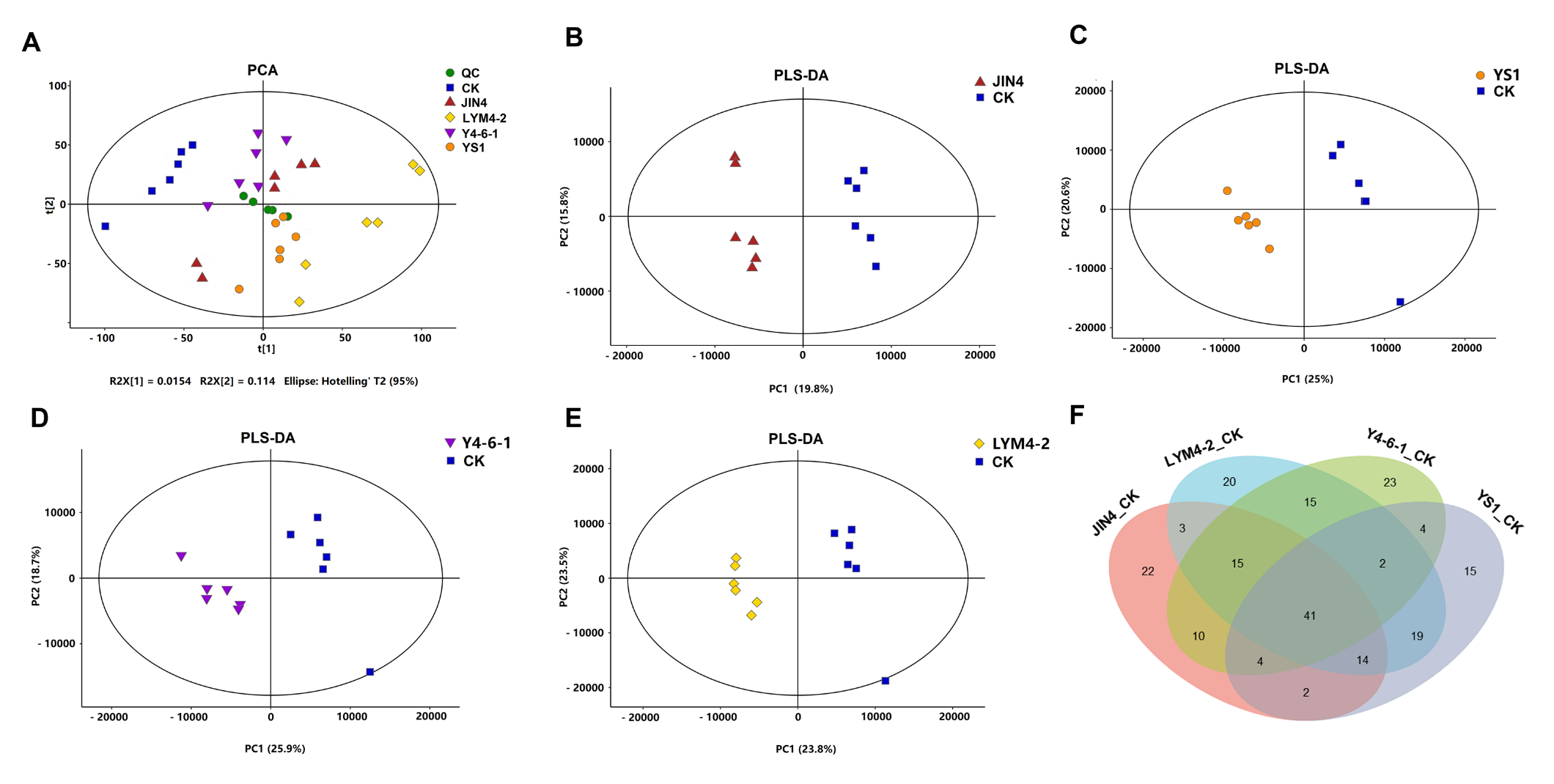


**Fig. S2.** Metabonomic analysis of four *B. velezensis*-treated samples. (A) Principal component analysis (PCA) of metabolites in the rhizosphere soil. (B–E) PLS-DA analysis of metabolites. (F) Analysis of co-expressed DMs in different treatment groups. Refer to Table S1 for detailed annotation information for all metabolites.

**
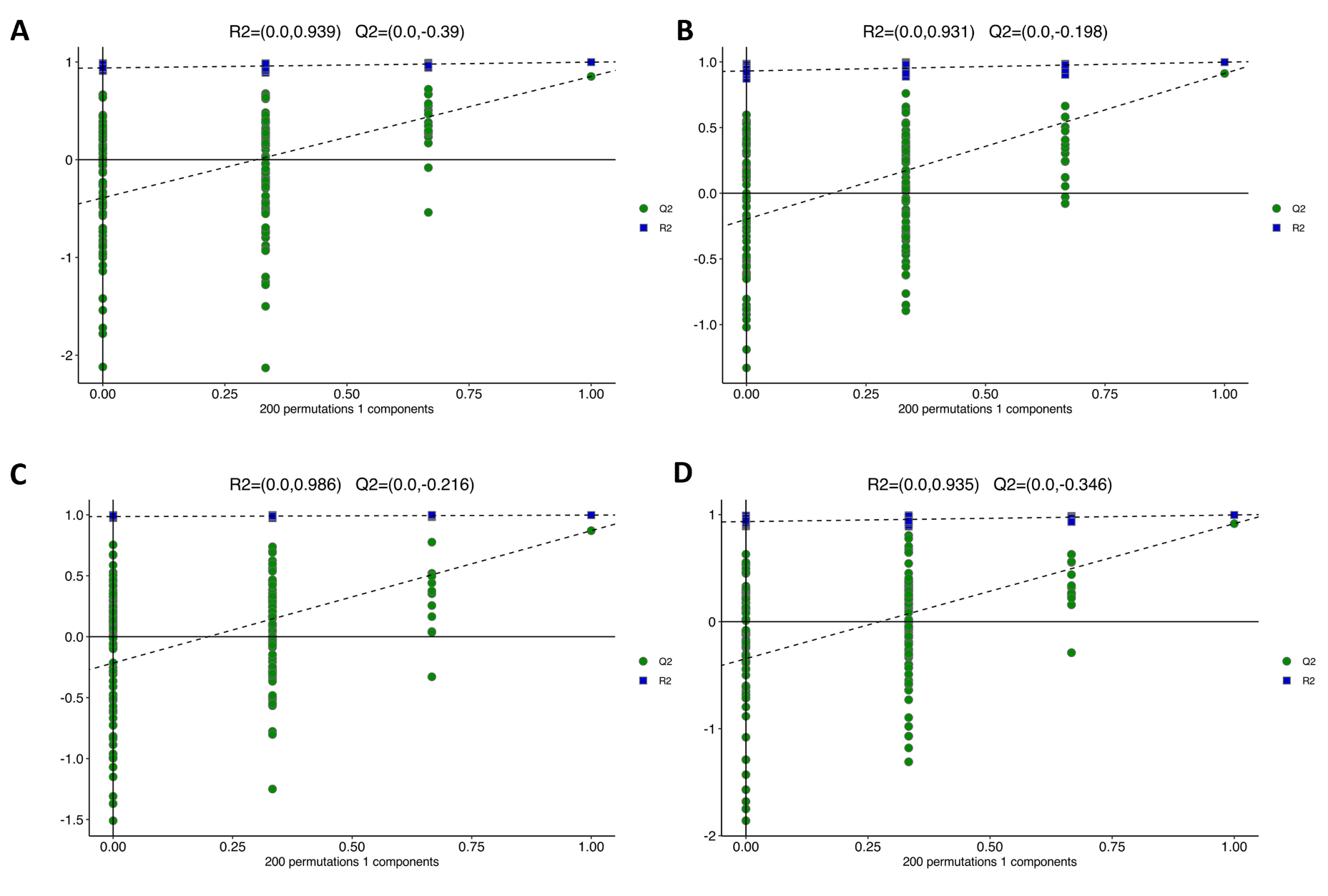
**

**Fig. S3.** Metabonomic analysis of four *B. velezensis* treated. (A-D) Cross-validation model of PLS-DA.


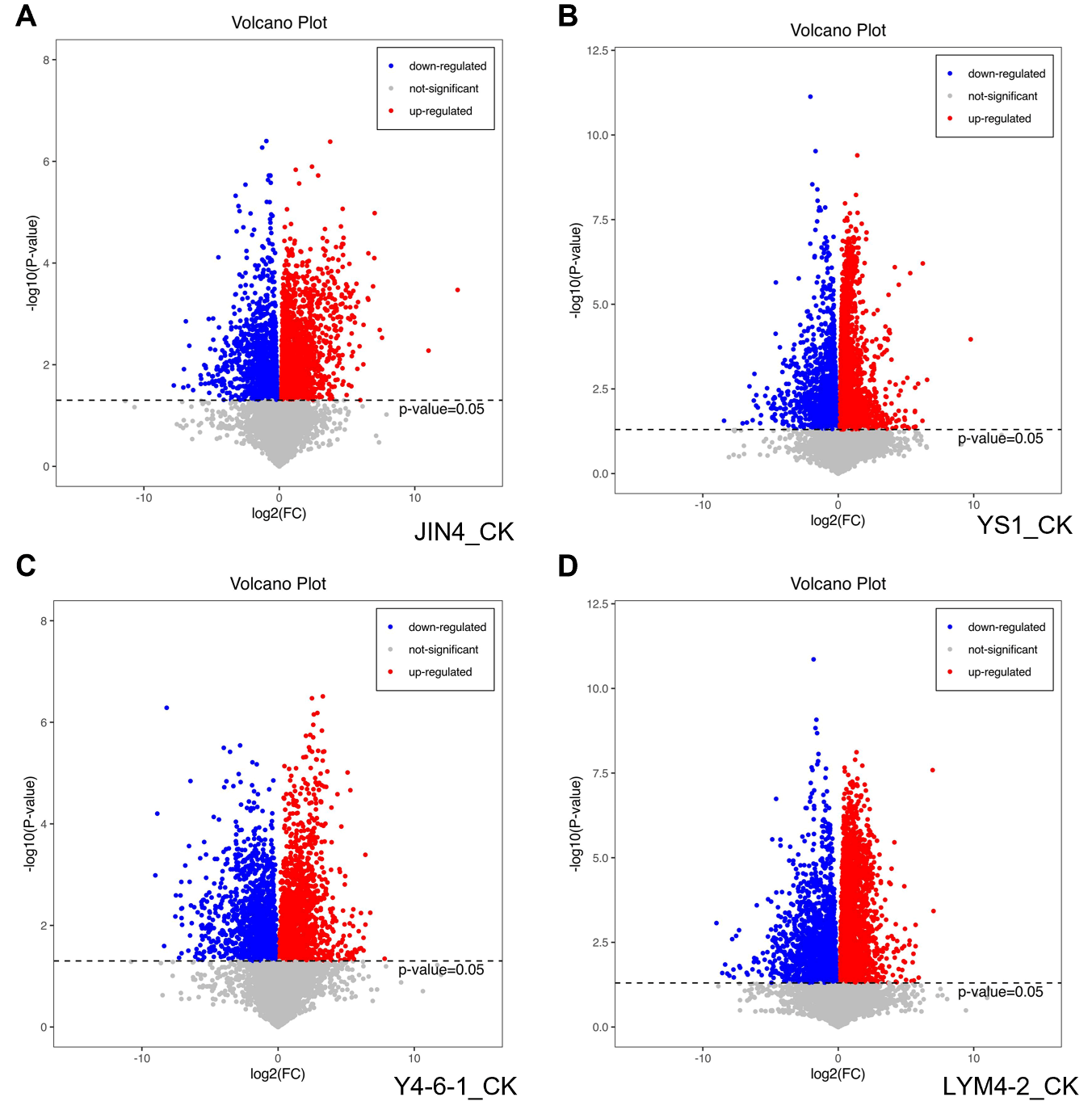


**Fig. S4.** The expression volcano map of differential metabolites up and down regulate. Blue dots represent down-regulated metabolites, red dots represent up-regulated metabolites, and gray dots represent no-differential metabolites.
